# Supplementary material for: Evolution of cooperation in multichannel games on multiplex networks
Source: PLoS Comput Biol. 2024 Dec 19;20(12):e1012678. doi: 10.1371/journal.pcbi.1012678 (PMC11698529; doi:10.1371/journal.pcbi.1012678)
Supplement: S1 Fig — Strategies players use (on an average) against their unique neighbors (panel a-b) and common neighbors (panel c-d) when the average fraction of common neighbors across two layers of the multiplex network are (a) 0, (b, d) 0.33, and (c) 1 respectively. O=0.33 indicates each individual has OiCN=k/2=10 common neighbors, where k = 20 is the degree of each node of the RRN in both layers of the multiplex network with N = 100 nodes per layer. Each bar represents the population’s average of pa1,a2α(paαα) against common (unique) neighbors in that respective layer, averaged over 100 independent realizations using independent strategy update, whereas dots represent 50 randomly sampled realizations of the simulation. Other parameter values used are the same as in Fig 7. (PDF) [file pcbi.1012678.s001.pdf]

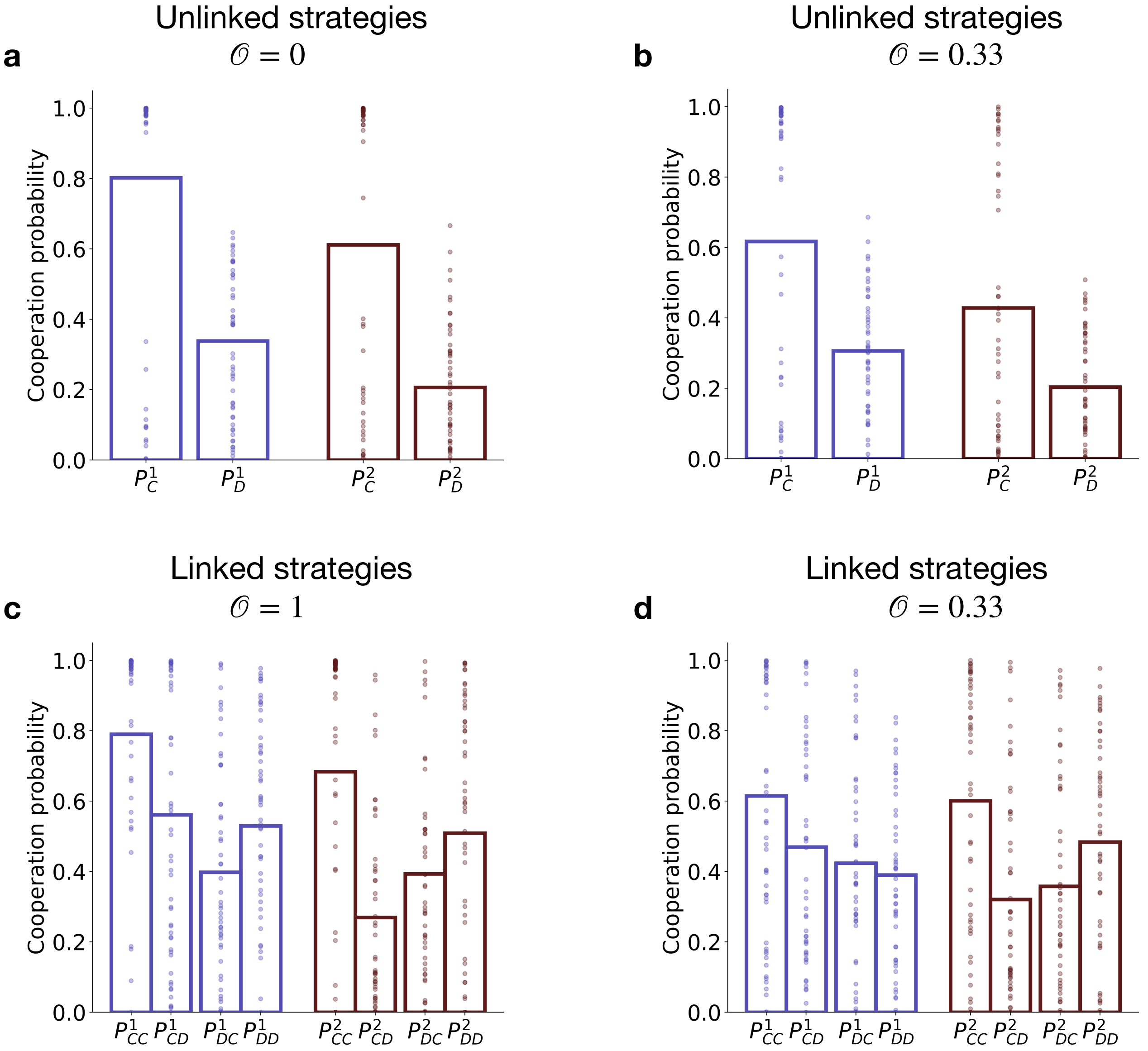

a
Unlinked strategies
𝒪 = 0
b
Unlinked strategies
𝒪 = 0.33
c
Linked strategies
𝒪 = 1
d
Linked strategies
𝒪 = 0.33
Cooperation probability
0.0
0.2
0.4
0.6
0.8
1.0
P_C^1 P_D^1 P_C^2 P_D^2
P_{CC}^1 P_{CD}^1 P_{DC}^1 P_{DD}^1 P_{CC}^2 P_{CD}^2 P_{DC}^2 P_{DD}^2
