# Supplementary figures and images for: Evolution of cooperation in multichannel games on multiplex networks
Source: PLoS Comput Biol. 2024 Dec 19;20(12):e1012678. doi: 10.1371/journal.pcbi.1012678 (PMC11698529; doi:10.1371/journal.pcbi.1012678)

**a**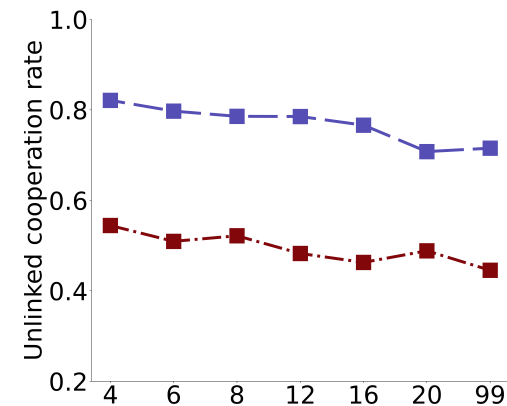**b**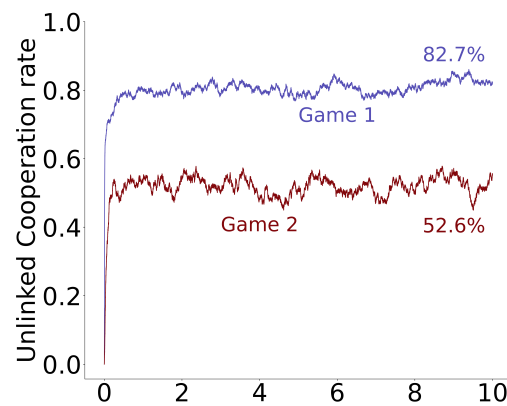**c**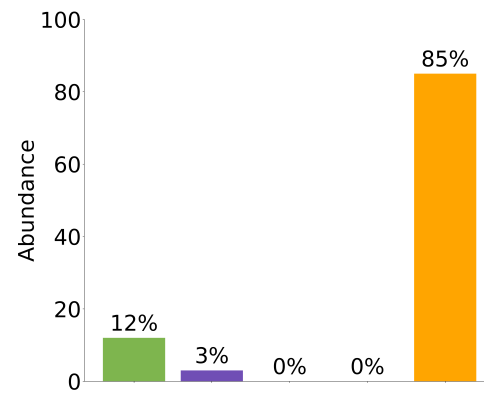**d**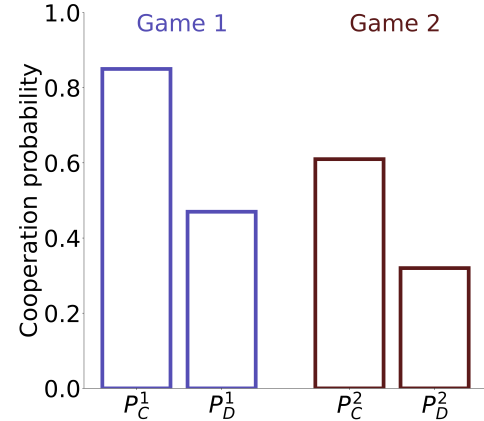**e**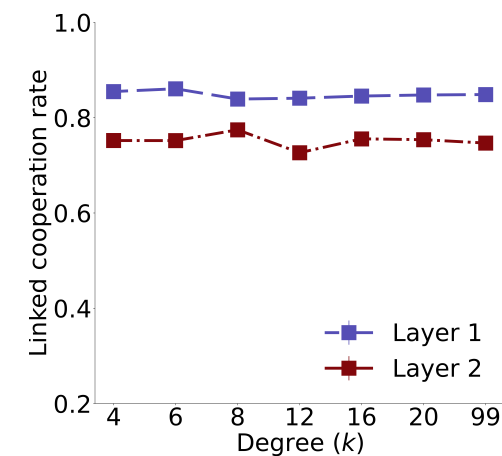**f**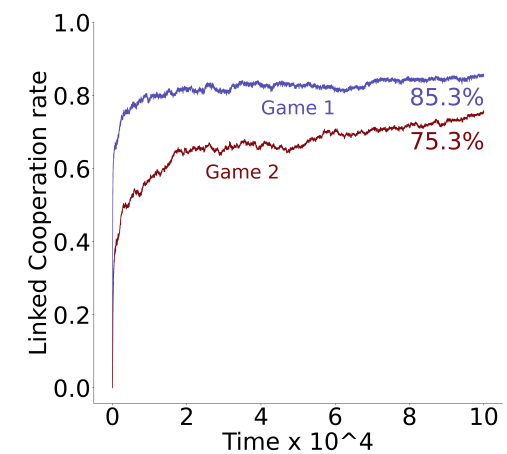**g**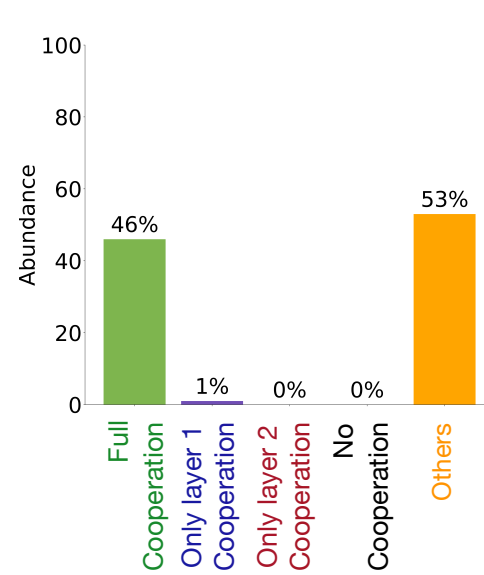**h**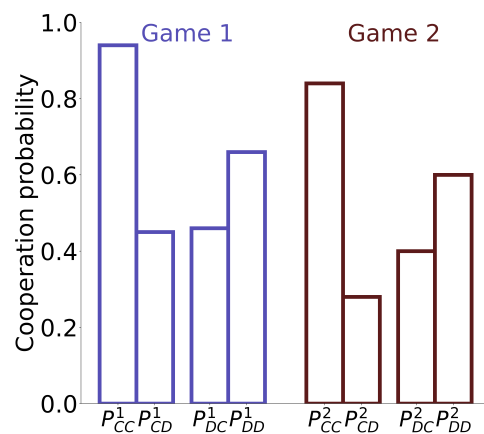

Supplement: S2 Fig — The Snowdrift game [70] is a version of the Chicken game, where defection is not an equilibrium of the game. In this game, it is always best to cooperate if the other individual defects and vice-versa. Using unlinked (a-d) and linked (e-h) strategies, we simulate the dynamics when players simultaneously engage in a game with larger benefit of cooperation (game 1, blue), and a game with relatively lower benefit of cooperation (game 2, brown) in layer 1 and layer 2 of a multiplex network respectively. (a,e) shows the variation of the cooperation rate with degree (k1 = k2 = k); (b,f) the time evolution of the cooperation rate for k = 4; (c,g) relative abundance of different cooperation scenarios across multiple simulations for k = 4; (d,h) the average strategy used for k = 4; when all individuals use unlinked and linked strategies respectively. The results were obtained by averaging over 100 simulations using the independent strategy update rule and RRN topology for each layer of the multiplex network. For Snowdrift games considered here, R1=b1-c2, S1 = b1 − c, T1 = b1, P1 = 0, and R2=b2-c2, S2 = b2 − c, T2 = b2, P2 = 0. Other parameters used: N = 100, b1 = 2.5, b2 = 1.2, c = 1, μ = 0.001, w = 1, and s = 2. (PDF) [file pcbi.1012678.s002.pdf]

**a**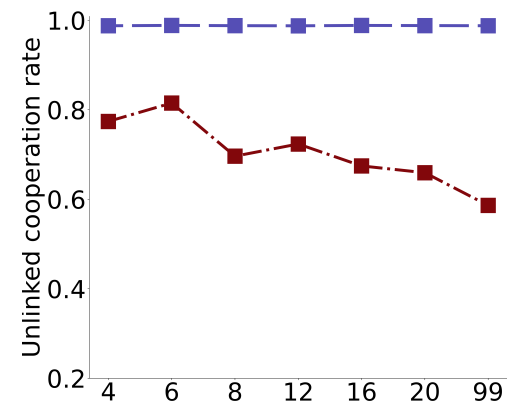**b**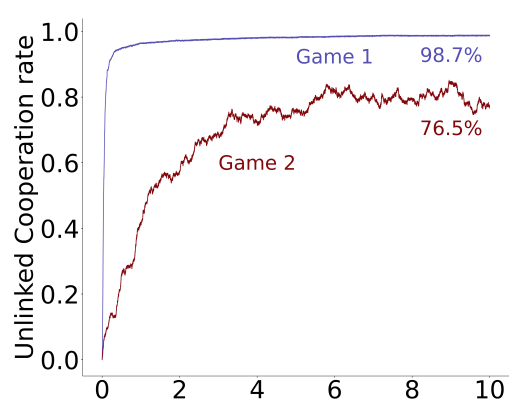**c**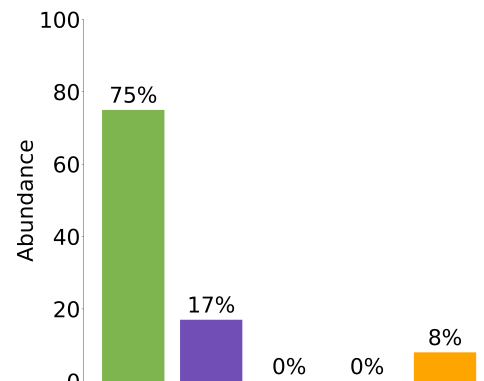**d**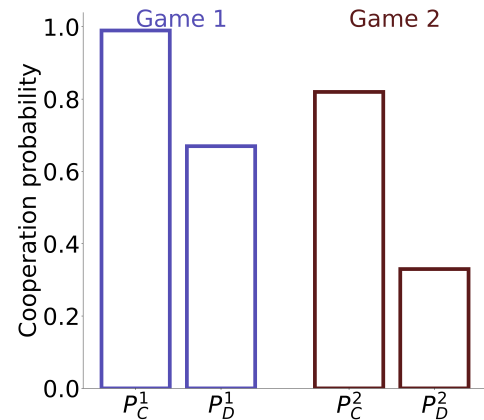**e**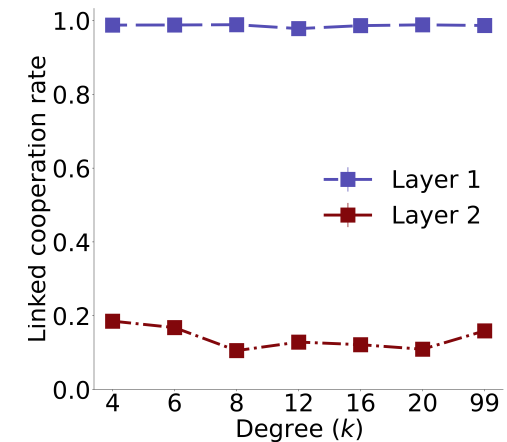**f**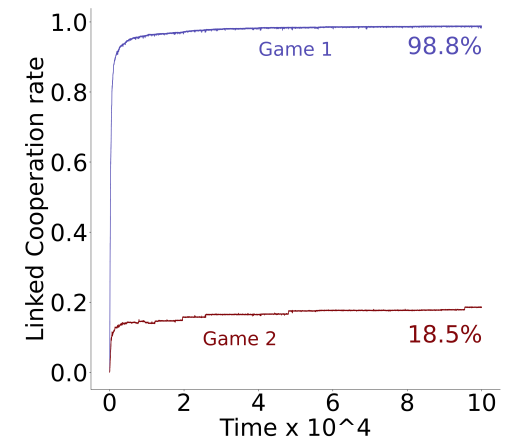**g**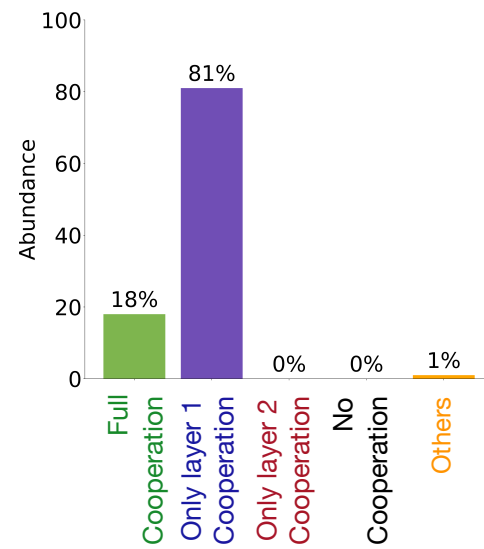**h**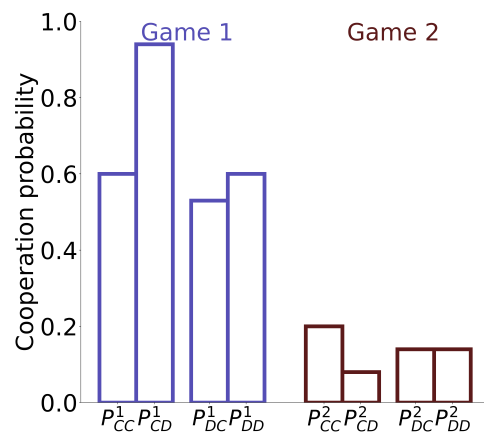

Supplement: S3 Fig — The Sculling game [71] is a variant of the Stag-Hunt game in which the benefits of cooperation depends non-linearly on the number of cooperators. For the selected parameters, this game falls within the coordination class, where mutual cooperation is an equilibrium even if the game is repeated only once. Using unlinked (a-d) and linked (e-h) strategies, we simulate the dynamics when players simultaneously engage in a game with larger benefit of cooperation (game 1, blue), and a game with relatively lower benefit of cooperation (game 2, brown) in layer 1 and layer 2 of a multiplex network respectively. (a,e) shows the variation of the cooperation rate with degree (k1 = k2 = k); (b,f) the time evolution of the cooperation rate for k = 4; (c,g) relative abundance of different cooperation scenarios across multiple simulations for k = 4; (d,h) the average strategy used for k = 4; when all individuals use unlinked and linked strategies respectively. The results were obtained by averaging over 100 simulations using the independent strategy update rule and RRN topology for each layer of the multiplex network. For Sculling games considered here, R1=43b1-c, S1=b13-c, T1=b13, P1 = 0, and R2=43b2-c, S2=b23-c, T2=b23, P2 = 0. Other parameters used: N = 100, b1 = 2.5, b2 = 1.2, c = 1, μ = 0.001, w = 1, and s = 2. (PDF) [file pcbi.1012678.s003.pdf]
