# Supplementary material for: Evolution of cooperation in multichannel games on multiplex networks
Source: PLoS Comput Biol. 2024 Dec 19;20(12):e1012678. doi: 10.1371/journal.pcbi.1012678 (PMC11698529; doi:10.1371/journal.pcbi.1012678)
Supplement: Attachment — Submitted filename: Response to Reviewers.pdf [file pcbi.1012678.s005.pdf]

Response to Reviewers

We thank both reviewers for their positive comments and constructive criticisms about our manuscript and we are glad to note that both reviewers found our work relevant and valuable. We have addressed all issues raised by the reviewers in our revised manuscript and have provided detailed responses to the reviewers' comments below. We hope that with these changes, the revised manuscript can be accepted for publication.

List of major changes

1. We have expanded the discussion on previous literature pertaining to games in multiplex networks and highlighted the ways in which our work differs from the existing literature in paragraphs 4, 5, 6 of the Introduction section, as suggested by reviewer 2.
2. We have mentioned the concept of universal dilemma strength and cited the relevant literature in the first paragraph of section 2.3, as suggested by reviewer 1.
3. We have also included a new sub-section 3.6 titled "*Other games*" where we present the results for Snowdrift (SD) and Stag-Hunt (SH) games. The associated figures are provided as supporting information figures S3 Fig. and S4 Fig. This generalizes our analysis, previously restricted to PD games, to other dilemmas.

Response to Reviewer 1

Comment: This work successfully establishes a model of new multiplex networks (Layers-1 and 2), where each plays different PD in terms of dilemma strength in each layer. One novel point in the authors' modeling is that, depending on whether a focal edge is unique in one of the networks or common (the edge exists in both layers), the focal player's offer (C or D), i.e., updating procedure is different. As Fig. 1 intelligibly explains, an offer on unique edge stochastically references to what the game opponent in the edge (single memory strategy), while, in the case of common edge, this reflection affects on both offers on the focal agent. I guess that no one has ever studied such a model. In this sense, I positively evaluate this work. The authors mainly delivered MAS results in visuals.
For example, Figs. 2 & 3 confirm that emerged cooperation in layer 1 is more enhanced than that of layer 2. Honestly speaking, to me, it's not specifically surprising, simply because the dilemma weakness (that is inverse to the dilemma strength, which is mentioned as below) at layer 1 is more than that at layer 2 due to b_1 = 5 > b_2 – 3 while c_1 = c_2 =1.

Response: Our key motivation was to understand how strategy linking in structured populations affects the average cooperation rate in both layers of the multiplex network. We also wanted to understand how the average cooperation rate depends on the structural properties of the network layers such as the degree of the network in each layer and the degree of structural overlap between the layers. We wish to emphasize that the surprising result here is not that the average cooperation rate in layer 1 is larger than that in layer 2, that is expected (as the reviewer points out) since game 1 is the higher benefit game. The high level of cooperation observed (~80%) in layer 2 in the scenario with strategy linking, even for networks with high degree, was the surprising result since that is to be contrasted with the unlinked strategy scenario where the cooperation rate in layer 2 drops significantly with increasing degree. Another unanticipated result that points to the significance of a structured multilayer population shows how even

without strategy linking, a high average cooperation rate is observed even in the low-benefit game and the full cooperation scenario dominates for networks with low to moderate degree. All these issues have been extensively discussed in Section 3.1 of our manuscript.

Comment: One suggestion relevant to the point above is that the authors should mention about the concept of universal dilemma strength, quantified by the Chicken-type dilemma; Dg' := (T - R) / (R – P) and Stag Hunt-type dilemma; Dr' := (P – S) / (R – P). And they should note that the Donor & Recipient (D & R) game (the authors called the donation game) is special because of the same amount of Dg' = Dr' = c / (b – c), which is appropriate for a typical Prisoner's Dilemma game. They are suggested to add those points in the introduction or model depiction section by referring to relevant literatures; (i) Relationship between dilemma occurrence and the existence of a weakly dominant strategy in a two-player symmetric game, BioSystems 90(1), 105-114, 2007, (ii) Universal scaling for the dilemma strength in evolutionary games, Physics of Life Reviews 14, 1-30, 2015, (iii) Scaling the phase- planes of social dilemma strengths shows game-class changes in the five rules governing the evolution of cooperation, Royal Society Open Science, 181085, 2018, (iv) Sociophysics Approach to Epidemics, Springer, 2021.

Response: We acknowledge that the Donation game is special because Dg'=Dr'=c/(b-c). We have mentioned this in sub-section 2.3 called *Payoff Calculation* under the **Methods** section and added the relevant references.

We have studied both the Chicken/Snowdrift (SD) game as well as the Stag-hunt (SH) game as suggested by the reviewer in order to understand how the different nature of the games played on the multiplex networks, exemplified by the nature of the dilemmas, affect our results. In the SD game, which is an anti-coordination game, linking of strategies can significantly increase the average cooperation rate and the abundance of full-cooperation scenarios in both layers in contrast to the case where strategies are unlinked. However, unlike in PD games, even though linking results in the increase in abundance of the full cooperation scenario in comparison to the unlinked case, the full cooperation scenario is not dominant even with strategy linking. In the SH game, the differences are even more striking, with linking being counterproductive to the spread of cooperation in layer 2 where the lower benefit game is being played.

In a new sub-section 3.6 titled *Other games*, under the **Results** section in the main text, we have highlighted these distinctions between the results of the PD games and other coordination (SH) and anti-coordination (SD) games. The figures associated with SD and SH games have been included in the supporting information figures (see S3 Fig. and S4 Fig.).

Response to Reviewer 2

Comment: The manuscript presents a model where individuals engage in Prisoner's Dilemma (PD) games within a multiplex network, with various layers representing different interaction contexts (different PD games). The main focus is on how cooperation evolves based on the network structure, specifically the edge overlap between layers, and how imperfections in memory influence strategies. This is indeed an interesting topic, as cooperation in complex networks remains a relevant issue in evolutionary game theory.

The use of real-world data, such as the interactions among Florentine families and employees at Aarhus University, adds significant value to the study. It effectively demonstrates how theoretical models can be applied to real-life situations, enriching the analysis of cooperation dynamics.

***Response*:** We thank the reviewer for these positive comments about our manuscript.

Comment: However, there are a few areas where the paper could be enhanced. While the approach is relevant, there are numerous studies addressing cooperation in multilayer and multiplex networks. The paper would benefit from a clearer articulation of what distinguishes this work from existing research. A more explicit comparison with previous models would help contextualize the contributions and highlight their uniqueness. Without this context, even readers and researchers interested in this field may find it challenging to assess the manuscript's originality; thus, contextualizing the results with prior research is essential.

***Response*:** We have added a couple of paragraphs to the Introduction summarising relevant literature associated with games on multiplex networks and highlighted the difference between the existing literature and our work.
